# Radio-Frequency Biosensors for Real-Time and Continuous Glucose Detection

**DOI:** 10.3390/s21051843

**Published:** 2021-03-06

**Authors:** Chorom Jang, Hee-Jo Lee, Jong-Gwan Yook

**Affiliations:** 1 Department of Electrical and Electronic Engineering, Yonsei University, Seoul 03722, Korea; chorom@yonsei.ac.kr; 2 Department of Physics Education, College of Education, Daegu University, Gyeongsan 38453, Korea; hjlee@daegu.ac.kr

**Keywords:** radio-frequency, microwave, insulin, glucose, real-time, biosensor, diabetes

## Abstract

This review paper focuses on radio-frequency (RF) biosensors for real-time and continuous glucose sensing reported in the literature, including our recent research. Diverse versions of glucose biosensors based on RF devices and circuits are briefly introduced, and their performances are compared. In addition, the limitations of the developed RF glucose biosensors are discussed. Finally, we present perspectives on state-of-art RF biosensing chips for point-of-care diagnosis and describe their future challenges.

## 1. Introduction

**Figure 1:**
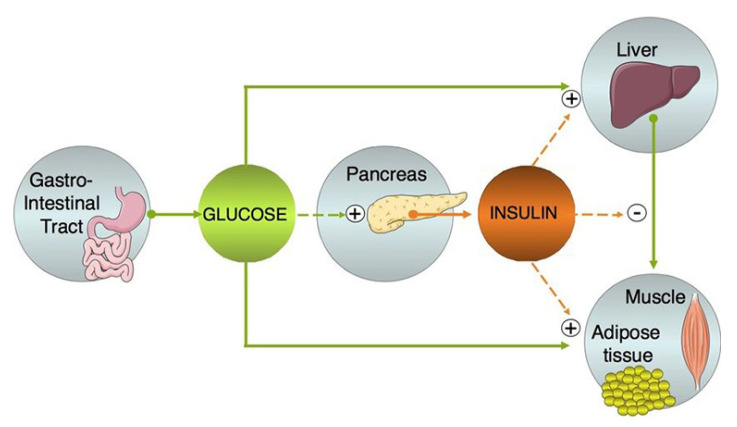
High-level representation of the glucose-insulin control system (Reprinted with permission from [4]; published by Mary Ann Liebert, Inc., New Rochelle, NY, USA, 2004).

Glucose is a type of sugar obtained from carbohydrates, such as bread, potatoes, and fruit, that the human body uses for energy [1]. Insulin is a hormone that moves glucose from the blood into the cells for energy and storage [2], as shown in Figure 1. In general, diabetes can be classified into two types: in type one diabetes, a person does not have sufficient insulin to move glucose through cells, causing the immune system to attack and destroy cells of the pancreas, and in type two diabetes, the cells do not respond to insulin as well as they should [3]. Thus, insulin plays a crucial role in regulating glucose homeostasis and, more specifically, in keeping the glucose concentration in the blood (glycemia) within a narrow range (0.8–1 g/L). Dysfunctions in the regulation of glucose homeostasis can lead to chronic hyperglycemia [4].

Hyperglycemia can be classified into postprandial hyperglycemia (over 180 mg/mL for 2 h after eating) and fasting hyperglycemia (over 130 mg/mL after not eating or drinking for 8 h). In the case of diabetic patients, their pancreas, where insulin is made, needs to make increasingly more insulin to move glucose into the cells. Eventually, the pancreas is damaged and cannot make sufficient insulin to meet the body’s needs. In addition, high blood glucose over a long period of time can damage the kidneys, eyes, and other organs [5,6].

For this reason, doses of insulin need to be administered, and it is vital that diabetic patients regularly monitor the level of glucose in their blood. With the currently available glucose biosensors, a patient himself or herself can extract a small drop of blood and obtain a direct digital readout of the glucose concentration within 1 min. Such glucose monitoring involves a prick test to extract the blood, which can be painful, especially for children. In severe cases, the repercussions can be fatal.

For the systematic administration of a patient’s glucose, continuous glucose monitoring (CGM) using minimally invasive sources such as sweat or tears is less demanding. CGM improves healthcare by providing a higher data collection rate with increased reliability while avoiding the discomfort of the prick test [7,8]. From this point of view, a real-time and continuous radio-frequency (RF) glucose biosensor with the great advantages of being noninvasive and noncontact and monitoring a nonionized RF spectrum can be a new alternative. With the motivation of designing such real-time and continuous glucose-diagnostic RF devices, many studies have focused on the characterization of the dielectric properties of glucose solutions. The possibility of using RF or microwave sensing for blood glucose level characterization has also been investigated. In recent years, with the development of wireless technologies, an increased interest in noninvasive, noncontact, and continuous RF glucose detection has emerged [9,10,11,12].

In this paper, we review the figures of merit of representative RF biosensors for noninvasive and continuous glucose detection, including our own sensing scheme, through case studies. In addition, we suggest improvements to robust RF biosensors and present related perspectives. This paper is organized as follows. We begin by introducing the historical landmarks of three generations of glucose sensors, the dielectric properties of glucose solutions in the RF spectrum, and the measurement of these properties using two types of RF schemes, i.e., antennas and resonators. We also briefly introduce the principles of glucose sensing using representative RF devices and circuits, such as cavity resonators, microprobes, substrate-integrated waveguides (SIWs), antennas, and planar resonators, and we evaluate the performance of the proposed glucose biosensors. We conclude with a brief discussion of possible ways to meet the sensitivity and selectivity challenges for practical application.

## 2. History of Glucose Sensors

### 2.1. Definition of a Biosensor and Its Components

A biosensor can be defined as a compact analytical device or unit incorporating biological or biologically derived sensitive recognition elements, e.g., receptors, enzymes, antibodies, nucleic acids, microorganisms, or lectins [13,14], integrated or associated with physicochemical transducers, such as those based on electrochemical, optical, thermometric, piezoelectric, or magnetic schemes [15,16]. Thus, a biosensor has three main parts: (i) a biological recognition element to detect the target biomolecules (analytes) when various substances are present, (ii) a transducer to convert the biomolecules detection phenomenon into a measurable electric signal, and (iii) a post-processing system to convert the electric signal into a legible form [17,18,19].

### 2.2. First-Generation (1st G) Glucose Biosensors

**Figure 2:**
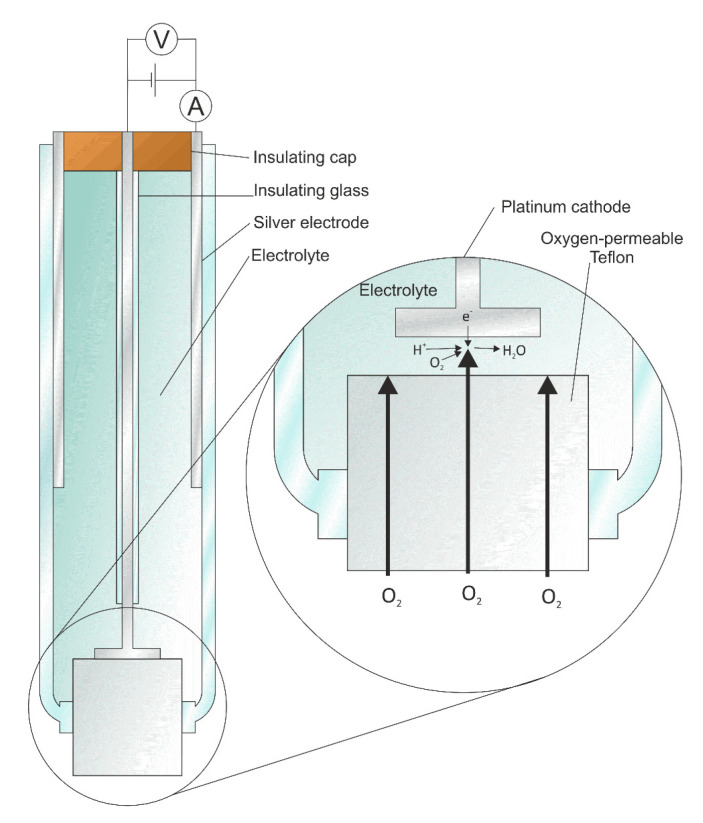
Schematic representation of the glucose biosensor invented by Clark and Lyons [20].

In 1962, Clark and Lyons invented the first biosensor for detecting glucose [17]. This sensor, called an oxygen electrode sensor, consisted of an oxygen electrode, an inner oxygen-semipermeable layer, a thin layer of GOx, and an outer dialysis membrane [20], as shown in Figure 2. This glucose biosensor was based on the following chemical reaction [21]:

(1) $$\begin{array}{c} \mathrm{Glucose}+O_{2}+H_{2}O\overset{GOD}{\to }\mathrm{glucose}\;\mathrm{acid}+H_{2}O_{2}. \end{array}$$

Here, the enzyme glucose oxidase (GOD) catalyzes the oxidation of glucose into gluconic acid. At the electrode:

(2) $$\begin{array}{c} O_{2}+2e^{-}+2H^{+}=H_{2}O_{2}. \end{array}$$

Updike and Hicks made a simplified glucose assay electrochemically through immobilization and stabilization of the GOx since they invented the first glucose biosensor in 1962 [18,22]. Also, they proposed the immobilization of GOx in a polyacrylamide gel on an oxygen electrode and measured the glucose concentration in a biofluid system [18]. In 1975, the first commercial glucose sensors using Clark’s method were initiated, and these sensors were based on amperometric detection of hydrogen peroxide. The peroxide formation could be easily measured in the context of miniature devices [23]. However, the main problem was that the amperometric measurement of hydrogen peroxide needs a high operation potential for enhancing selectivity [24].

### 2.3. Second-Generation (2nd G) Glucose Biosensors

During the 1980s, glucose biosensors became a popular topic of research, reflecting the growing emphasis on biotechnology. To overcome the limitations of first-generation glucose biosensors, redox mediators carrying electrons from the enzyme to the surface of the working electrode [25] were introduced. During this decade, intense efforts were focused on developing redox-mediator-based glucose biosensors [26,27], called second-generation glucose biosensors. Thus, a variety of electron mediators, such as ferrocene, ferricyanide, quinine, tetrathiafulvalene (TTF), tetracyanoquinodimethane (TCNQ), thionine, methylene blue, and methyl viologen, were used to improve sensor performance [26,27,28,29]. In addition, various techniques to promote electron transfer between the GOx redox center and the electrode surface were used, such as enzyme wiring of GOx with electron-conducting redox hydrogels, the chemical modification of GOx using electron-relay groups, and the application of nanomaterials as electrical connectors [23,30,31,32].

### 2.4. Third-Generation (3rd G) Glucose Biosensors

**Figure 3:**
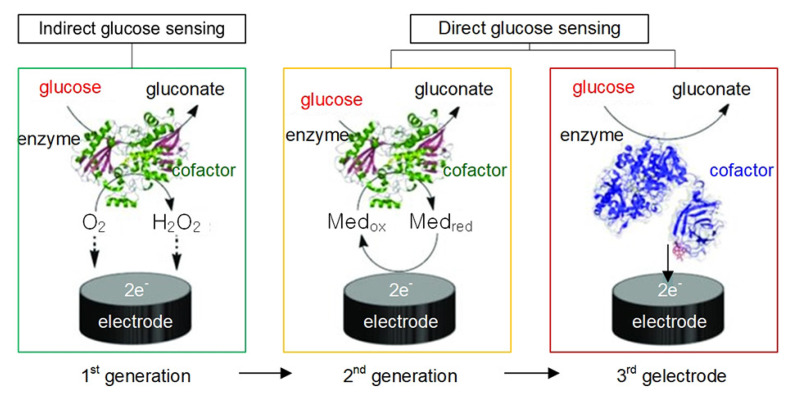
First-, second-, and third-generation glucose biosensors.

In the 1990s, third-generation glucose biosensors emerged, which were reagentless and based on direct transfer between the enzyme and the electrode without mediators. To avoid the need for highly toxic mediators, the electrode can perform direct electron transfer using organic conducting materials based on charge-transfer complexes [33,34], as shown in Figure 3. As a result, third-generation glucose biosensors have led to implantable, needle-type devices for continuous in vivo monitoring of blood glucose. The absence of mediators also endows these biosensors with superior selectivity.

As described above, the majority of glucose biosensors developed over the last few decades have been of the electrochemical type because of their better sensitivity, higher reproducibility, ease of maintenance, and low cost. The major historical landmarks in the development of electrochemical glucose biosensors are summarized in Table 1.

## 3. Radio-Frequency Characteristics of Glucose

### 3.1. Dielectric Properties of Glucose

Dielectric properties are critical factors in gaining a better understanding of the interactions of microwaves with aqueous glucose solutions. The dielectric properties of a material are defined in terms of its dielectric constant ($\varepsilon^{\prime }$) and loss factor ($\varepsilon^{\prime \prime }$). $\varepsilon^{\prime }$ is a measure of the ability of a material to couple with microwave energy, and $\varepsilon^{\prime \prime }$ is a measure of the ability of a material to be heated by absorbing microwave energy [37].

#### Cole-Cole Model of the Dielectric Constant of an Aqueous Glucose Solution

**Figure 4:**
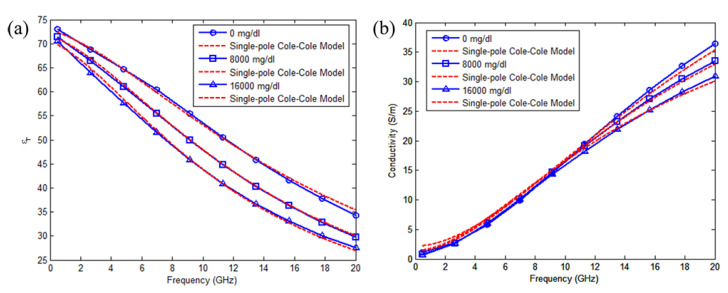
(**a**) Dielectric constant and (**b**) conductivity values obtained form measured data and corresponding fitted models at three differential glucose levels in the 0.5–20 GHz band (Reprinted with permission from [44]; published by IEEE, Toulouse, France, 2011).

The Cole-Cole model offers an efficient and accurate representation of many types of biological tissues over a very broad frequency band and has been recently used to reduce the complexity of the experimental data obtained for various human tissues, such as brain, fat, breast, skin, bone, and liver tissues [38,39,40,41,42]. A Cole-Cole fitting model for the wideband dielectric properties of aqueous solutions of various glucose concentrations has been proposed as follows [43]:

(3) $$\begin{array}{c} \hat{\varepsilon }\left(\omega \right)=\varepsilon_{c}^{\prime }\left(\omega \right)-j\varepsilon_{c}^{\prime \prime }\left(\omega \right)=\varepsilon_{\infty }+\sum_{n}\frac{\varepsilon_{s}-\varepsilon_{\infty }}{1+{\left(j\omega \tau_{n}\right)}^{\left(1-\alpha_{n}\right)}}+\frac{\sigma_{i}}{j\omega \varepsilon_{0}}, \end{array}$$

where ω is the angular frequency, $\varepsilon_{c}^{\prime }\left(\omega \right)$ is the dielectric constant as a function of frequency, $\varepsilon_{c}^{\prime \prime }\left(\omega \right)$ is the dielectric loss as a function of frequency, $\varepsilon_{s}$ is the static permittivity, $\varepsilon_{\infty }$ is the permittivity at high frequency, *n* is the order of the Cole-Cole fitting model, $\tau_{n}$ is the relaxation time, $\alpha_{n}$ is a coefficient representing the broadness of relaxation time distribution with value of 0 ≤ $\alpha_{n}$ ≤ 1, and $\sigma_{i}$ is the electrical conductivity. Based on the fitting formula given in (3), the real and imaginary permittivities at different concentrations are shown in Figure 4.

### 3.2. Dielectric Measurements of Aqueous Glucose Solutions

Recently, the applicability of microwave for noninvasive and continuous glucose monitoring has led to the investigation of the dielectric properties of blood and other liquids as a function of glucose concentration. For this purpose, the dielectric properties of blood [45], blood plasma [43], saline solutions [46], and deionized water [47] have been presented recent decades. Additionally, samples with various glucose levels have been measured using diverse RF measurement systems, such as antennas and resonators, as summarized in Table 2.

## 4. Radio-Frequency/Microwave-Based Glucose Sensors

### 4.1. Cavity-Type Glucose Sensors

Microwave cavity perturbation theory is widely used to determine the electrical properties of materials with high accuracy [58]. If a dielectric material is located in a microwave cavity, the resonant frequency will be perturbed [59]. Because glucose solutions of different concentrations have distinct electrical properties, the glucose concentration can be estimated from the change in the cavity characteristics by using the microwave cavity perturbation method.

Based on Maxwell’s equations and microwave cavity perturbation theory [60,61], the shift in the resonant frequency of a microwave cavity due to the presence of material in the cavity is expressed as follows:

(4) $$\begin{array}{c} \frac{f_{m}-f_{0}}{f_{0}}=\frac{-\;\int \int \int \;[\Delta \varepsilon \cdot {\left|E_{0}\right|}^{2}+\Delta \mu \cdot {\left|H_{0}\right|}^{2}]\;dV}{\;\int \int \int \;[\varepsilon \cdot {\left|E_{0}\right|}^{2}+\mu \cdot {\left|H_{0}\right|}^{2}]\;dV}, \end{array}$$

where $f_{m}$ and $f_{0}$ are the measured frequency and the initial resonant frequency, respectively; ε and μ represent the initial states of permittivity and permeability, respectively; Δε and Δμ are the variations in permittivity and permeability, respectively; and $E_{0}$ and $H_{0}$ represent the electric field intensity and magnetic field intensity, respectively.

At the resonant frequency, the time-averaged stored electric energy and magnetic energy are equal, and under the assumption that the dielectric material is located at the maximum electric field intensity, the resonant frequency shift can be simplified as follows:

(5) $$\begin{array}{c} \frac{f_{m}-f_{0}}{f_{0}}=\frac{-\;\int \int \int \;\;\Delta \varepsilon \cdot {\left|E_{0}\right|}^{2}\;dV}{2\;\int \int \int \;\;\varepsilon \cdot {\left|E_{0}\right|}^{2}\;dV}. \end{array}$$

**Figure 5:**
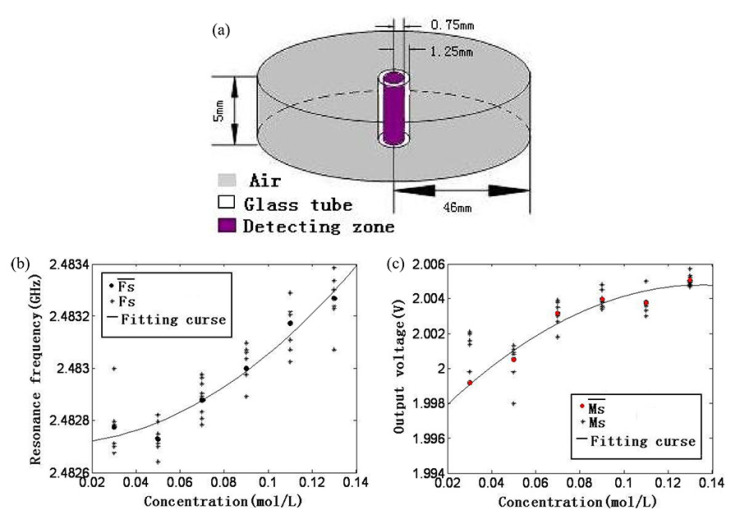
Glucose concentration detection using a $TM_{010}$ mode cylindrical cavity. (**a**) Configuration of the sensor. (**b**) Frequency shift depending on the glucose concentration. (**c**) Variation in the output voltage depending on the glucose concentration (Reprinted with permission from [63]; © 2021 IEEE).

Wang et al. proposed a glucose sensor based on a $TM_{010}$ mode microwave cavity with a resonant frequency of 3 GHz combined with a digital signal processing (DSP) block and a microwave power detector [62]. The DSP block generates a microwave sweep signal from DC to 3 GHz for input to the cavity as a controller. Additionally, it samples and analyzes the output signal of the cavity through the microwave power detector as a signal processor. Glucose solutions with different concentrations of 50, 90, and 120 mmol/L were placed at the location of the maximum electric field intensity, and the sensor detected them based on the variations in the frequency shift and the amplitude of the output power. Both the resonant frequency and amplitude increased as the concentration of the glucose solution increased. Fan et al. proposed a glucose sensor consisting of a $TM_{010}$ mode cylindrical cavity, as shown in Figure 5a, which resonates at 3 GHz; a microwave scanning generator; a detection circuit; an A/D converter; and a D/A converter [63]. The electric field of the microwave cylindrical cavity reaches its maximum at the center of the cavity. Thus, the glass tube for loading glucose solutions with different concentrations of 30, 50, 70, 90, 110, and 130 mmol/L was also placed at the center. Both the resonant frequency of the cavity and the output voltage of the detection circuit were demonstrated to increase with an increasing glucose concentration, as shown in Figure 5b,c.

Li et al. measured glucose concentrations from 70 mg/dL to 150 mg/dL in steps of 10 mg/dL using a $TE_{011}$ mode microwave cavity [64]. The $TE_{011}$ mode was selected due to its low loss and high Q-factor. The cavity is coupled with a waveguide through a small aperture to assemble a measurement system. The resonant frequency of the glucose sensor is 7.542 GHz. A capillary tube containing the glucose solution penetrates the center of the cavity. The measurement results showed that the resonant frequency increases linearly as the glucose concentration increases.

### 4.2. Microprobe-Type Glucose Sensors

The glucose concentration of a sample can also be detected from the changes in the reflection coefficient $S_{11}$ and transmission coefficient $S_{21}$ of a microwave cavity using a near-field microwave microprobe (NFMM) [65]. Based on standard transmission line theory, the reflection coefficient is expressed as follows:

(6) $$\begin{array}{c} S_{11}=20\log_{10}\left|\frac{Z_{in}-Z_{0}}{Z_{in}+Z_{0}}\right|, \end{array}$$

where $Z_{in}$ is the complex input impedance of the sensor and $Z_{0}$ is the complex impedance of the microstrip line, which is typically 50 Ω. The value of $Z_{in}$ depends on the permittivity; thus, a change in the glucose concentration causes a change in $S_{11}$.

**Figure 6:**
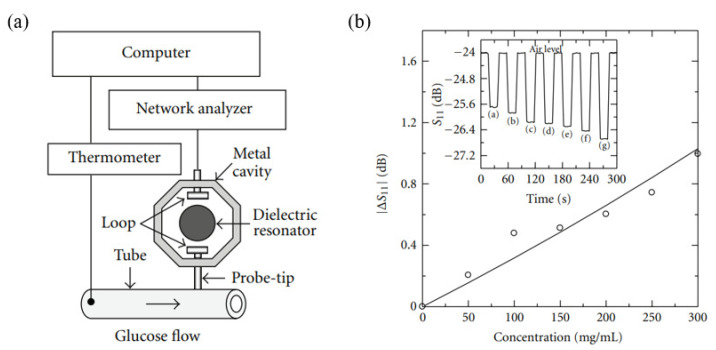
Glucose concentration detection using a $TM_{011}$ mode metal cavity and a near-field microprobe. (**a**) Experimental setup for glucose concentration detection. (**b**) Change in reflection coefficient hias a function of the glucose concentration. The inset represents a real-time diagram of the reflection coefficient for different DI water concentrations to 300 mg/mL. ((a) DI water, (b) 50 mg/mL, (c) 100 mg/mL, (d) 150 mg/mL, (e) 200 mg/mL, (f) 250 mg/mL, and (g) 300 mg/mL) [66].

Bababjanyan et al. measured glucose concentrations in a range of 0 to 300 mg/mL from the changes in the reflection coefficient [66]. The sensor consisted of a high Q-factor dielectric resonator, a $TE_{011}$ mode metal cavity with an Al probe tip, and a silicone tube containing the glucose solution, as shown in Figure 6a. The resonant frequency of the sensor was 4.6 GHz, and the unloaded Q-factor was 24,000. The sensor can be analyzed based on an equivalent impedance model of the probe-sample configuration. For this sensor, the complex input impedance is expressed as follows:

(7) $$\begin{array}{c} Z_{in}≅jZ_{a}k_{a}\frac{\left(2t_{g}+t_{t}\right)-k_{a}^{2}t_{t}t_{g}^{2}\varepsilon_{g}}{1-k_{a}^{2}t_{g}\varepsilon_{t}\left(t_{g}+t_{t}\right)-k_{a}^{2}t_{t}t_{g}\varepsilon_{g}}, \end{array}$$

where $Z_{a}$ is the complex impedance of air (377 Ω); $k_{a}$ is the wave vector in air; $t_{g}$ and $t_{t}$ are the wall thickness and diameter, respectively, of the cylindrical silicon tube; and $\varepsilon_{t}$ and $\varepsilon_{g}$ are the permittivities of the silicon tube and the glucose solution, respectively. As the glucose concentration increases, the reflection coefficient increases, as shown in Figure 6b.

Lee et al. proposed a glucose sensor based on a dielectric resonator, a $TE_{011}$ mode cavity, and a microwave microprobe [67]. The resonant frequency of this sensor is 4.5 GHz. The change in the reflection coefficient was used to detect glucose solutions with different concentrations from 0 to 10 mg/mL. The complex input impedance is written as follows:

(8) $$\begin{array}{c} Z_{in}=\frac{Z_{a}}{\sqrt{\varepsilon_{s}}}\cdot \frac{\left(Z_{a}/\sqrt{\varepsilon_{g}}\right)+j\left(Z_{a}/\sqrt{\varepsilon_{s}}\right)\tan\left(k_{a}t_{s}\sqrt{\varepsilon_{s}}\right)}{\left(Z_{a}/\sqrt{\varepsilon_{s}}\right)+j\left(Z_{a}/\sqrt{\varepsilon_{g}}\right)\tan\left(k_{a}t_{s}\sqrt{\varepsilon_{s}}\right)}, \end{array}$$

where $Z_{a}$ is the complex impedance of free space, $k_{a}$ is the wave vector of free space, $t_{s}$ is the thickness of the glucose solution, and $\varepsilon_{s}$ and $\varepsilon_{g}$ are the relative permittivities of the glucose solution and the glass substrate, respectively. The sensor response was analyzed according to the sample volume. The reflection coefficient of the sensor increases with increasing sample volume but saturates at approximately 50 μL. The measurement results showed that the reflection coefficient also increases with an increasing glucose concentration.

**Figure 7:**
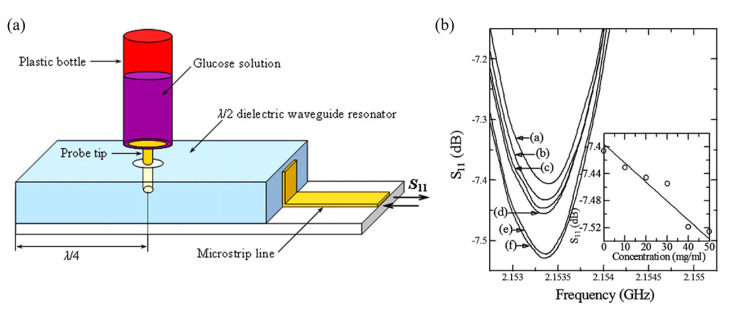
Glucose concentration detection using a $TM_{011}$ mode metal cavity and a near−field microprobe. (**a**) Configuration of the sensor. (**b**) Variation in the reflection coefficient as a function of the glucose concentration. ((a) DI water, (b) 10 mg/mL, (c) 20 mg/mL, (d) 30 mg/mL, (e) 40 mg/mL and (f) 50 mg/mL) (Reprinted with permission from [68]; published by Elsevier, 2009).

Kim et al. measured glucose solutions of 0–50 mg/mL using a microwave dielectric waveguide probe [68]. The probe-based sensor consisted of a half-wavelength dielectric resonator coupled with a microstrip line, and the resonant frequency was 2.15 GHz, as shown in Figure 7a. The complex input impedance of the sensor is expressed as follows:

(9) $$\begin{array}{c} Z_{in}=Z_{a}\cdot \frac{1+k_{a}^{2}\varepsilon_{g}V_{g}^{2}/S_{g}^{2}}{k_{a}^{2}\varepsilon_{g}^{2}V_{g}^{2}/S_{g}^{2}}+jZ_{a}\cdot \frac{1-k_{a}^{2}t_{s}\varepsilon_{g}V_{g}/S_{g}}{k_{a}\varepsilon_{g}V_{g}/S_{g}}, \end{array}$$

where $Z_{a}$ is the complex impedance of free space, $k_{a}$ is the wave vector in air, $t_{s}$ is the thickness of the substrate, $\varepsilon_{g}$ is the relative permittivity of the glucose solution, and $V_{g}$ and $S_{g}$ are the volume and surface area of the glucose solution, respectively. As the glucose concentration increases, the reflection coefficient decreases, as shown in Figure 7b.

### 4.3. SIW-Type Glucose Sensors

Nonplanar geometrical designs present limitations in terms of the complexity of fabrication, the high manufacturing cost, and the difficulty of achieving a sufficient sample volume. The sensor is immersed in the liquid sample to permit interaction between the electromagnetic waves and the dielectric properties of the sample [69]. Substrate-integrated waveguides (SIWs) are widely used for liquid characterization because of their high Q-factor with low measurement errors, low cost and straightforward design [70,71]. The principle of the frequency shift when using an SIW is developed from the conventional cavity perturbation method. However, there is one difference in terms of the dielectric permittivity [72]. In the conventional cavity perturbation method, the permittivity of air is used, whereas in the case of an SIW, the effective permittivity of the dielectric substrate is used. Thus, the frequency shift of an SIW is given as follows:

(10) $$\begin{array}{c} \frac{\Delta f}{f_{0}}=-\frac{\varepsilon -\varepsilon_{s}}{2\varepsilon_{s}}\cdot \frac{{\;\int \int \int \;}_{V_{s}}\;E_{0}^{*}\cdot E\;dV}{{\;\int \int \int \;}_{V_{c}}\;E^{2}\;dV}, \end{array}$$

where the left-hand side represents the fractional change in the resonant frequency due to the introduction of the sample into the cavity; ε and $\varepsilon_{s}$ are the complex permittivities of the sample and substrate, respectively; $V_{s}$ and $V_{c}$ represent the volumes of the sample and cavity, respectively; and $E_{0}$ and *E* are the initial and perturbed electric fields of the cavity, respectively. Kiani et al. proposed a planar SIW-type glucose sensor [73]. A hexagonal slot is etched on the top plane of the SIW, and a modified split-ring resonator (SRR) is connected to the slot for the planar sensing region. To focus the electric field on the sensing spot, two curved slots are added to the top plane of the sensor. In terms of transmission characteristics, the sensor acts as a bandstop filter. The resonant frequency of the sensor is 5.8 GHz, and the unloaded Q-factor is 130. Glucose solutions with different concentrations of 105, 255, 400, and 500 mg/dL were detected based on the fractional change in the resonant frequency. When the glucose concentration increases, the resonant frequency also increases.

**Figure 8:**
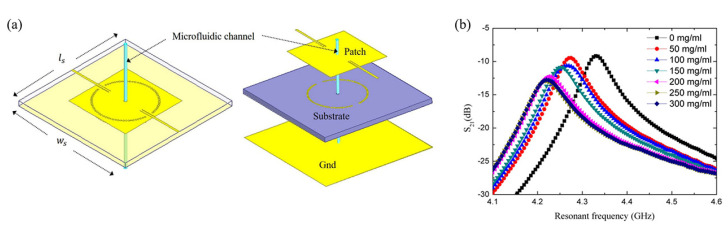
Glucose concentration detection using a $TM_{010}$ mode circular SIW with a microfluidic subsystem. (**a**) Configuration of the sensor. (**b**) Variation in the transmission coefficient with the glucose concentration [75].

A planar SIW has advantages of a planar resonator, such as being compact, cost-effective, and more straightforward to fabricate than a cavity, but a large sample volume is still required [74]. To solve this problem, a 2-port $TM_{010}$ circular SIW with a microfluidic subsystem for glucose concentration detection has been proposed [75]. The resonant frequency of the circular SIW is 4.4 GHz, and the transmission coefficient and Q-factor at the resonant frequency are −4.63 dB and 419, respectively. The electromagnetic field is strongest at the center of the circular SIW, so the sensing area is located at the center of the sensor. The microfluidic subsystem consists of a glass capillary and a channel slot, and this subsystem is transversely located at the center of the circular SIW where the electromagnetic near field is concentrated, as shown in Figure 8a. As an experimental demonstration, glucose solutions with different concentrations ranging from 0 mg/mL to 300 mg/mL in increments of 50 mg/mL were flowed through the glass capillary. The experimental results showed that both the resonant frequency and transmission coefficient decrease as the glucose concentration increases, as shown in Figure 8b.

### 4.4. Antenna-Type Glucose Sensors

The measurements using cavities or SIWs described in the previous subsections have limited potential for practical application to the human body because the sample needs to pass through the sensor. When using an antenna, the glucose concentration can be estimated without the need for the sample to penetrate into the sensor. Among the various candidate antenna structures, patch antennas are often used as sensors despite their narrow bandwidth because they are easy to design and fabricate [76,77,78]. For an arbitrary antenna, the boundary condition between the near field and the far field is as follows:

(11) $$\begin{array}{c} R=\frac{2D^{2}}{\lambda }, \end{array}$$

where *D* is the maximum physical dimension of the antenna and λ is the antenna wavelength. In the near-field region, the field impedances of the electric and magnetic dipoles differ significantly; specifically, the electric dipole has a very high field impedance, but the magnetic dipole has a low field impedance of approximately 0 Ω [79]. Thus, the change in permittivity due to a change in glucose concentration can affect the near-field antenna coupling [80]. Based on these properties, several studies on glucose concentration detection using patch antennas operating in the near-field zone have been conducted in recent decades.

A glucose sensor based on two matched antennas has been proposed [80]. The sensor consists of a patch antenna and an SIW slot antenna. The sensor has been optimized by considering the dielectric and mounting properties of human tissues. Glucose solutions of different concentrations ranging from 50 mg/dL to 500 mg/dL in increments of 50 mg/dL were detected based on the frequency shift and transmission coefficient at 5.5 GHz. The results showed that both the frequency and the transmission coefficient linearly decrease as the glucose concentration increases. Xiao et al. presented measurements of glucose solutions from 0 mg/dL to 4000 mg/dL using a UWB microwave detection technique [81]. One pair of microstrip antennas was located on both sides of the human earlobe. To accurately estimate the glucose concentration, the short-time Fourier transform (STFT) was used. In measurements with the sensor attached to an earlobe phantom, the transmission coefficient decreased linearly for 0–4000 mg/dL glucose solutions at 6.5 GHz.

**Figure 9:**
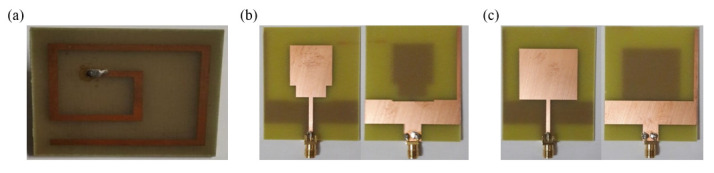
Three microstrip antennas compared in terms of their sensor performance for glucose detection. (**a**) Spiral antenna. (**b**) UWB patch antenna. (**c**) Narrowband patch antenna (Reprinted with permission from [84]; © 2021 IEEE).

Glucose concentration detection has also been conducted based on the variation in the reflection coefficient of a U-shaped microwave antenna [82]. The minimum reflection coefficient of the unloaded U-shaped antenna was observed at 1.9 GHz. For 0–20 mg/mL glucose solutions, the electromagnetic field distribution of the antenna was analyzed. When the sensing area of the antenna was immersed in 40 mL glucose solutions of different concentrations from 0 to 40 mg/mL, the reflection coefficient decreased. Costanzo designed an inset-fed microstrip patch antenna for glucose detection [83]. The target frequency of the proposed antenna is 2.4 GHz. Dielectric data of glucose solutions from 100 mg/dL to 500 mg/dL were collected using a standard open-ended coaxial probe. Based on these data, a simulation was conducted with respect to the loss tangent variation as a function of the glucose concentration. The experimental results showed that the reflection coefficient of the inset-fed microstrip antenna decreases with an increasing glucose concentration, whereas the resonant frequency increases. Deshmukh et al. designed three microstrip antennas for glucose concentration detection [84]: the spiral antenna shown in Figure 9a, the UWB patch antenna shown in Figure 9b, and the narrowband patch antenna shown in Figure 9c. The center frequencies of the three antennas are 4.69 GHz, 3.6 GHz, and 1.357 GHz, respectively. For glucose solutions with concentrations from 100 mg/dL to 350 mg/dL, the frequency shifts of the three microstrip antennas were compared, and the narrowband patch antenna was found to be the most sensitive among the three antennas.

**Figure 10:**
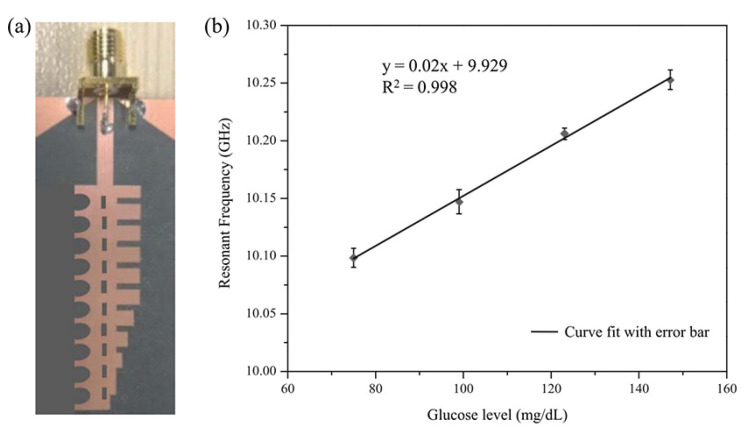
Glucose concentration detection using an SSPP endfire sensor. (**a**) Configuration of the sensor. (**b**) Change in the resonant frequency with the glucose concentration from 70 mg/dL to 150 mg/dL (Reprinted with permission from [85]; © 2021 IEEE).

Vrba et al. designed a sensor consisting of an inset-fed microstrip patch antenna and a small rectangular container on the top surface of the antenna [51]. They prepared two different types of liquid phantoms, consisting of physiological saline-glucose solutions and pig blood-glucose solutions with concentrations ranging from 0 mg/dL to 500 mg/dL, and the frequency shifts for the two types of liquid phantoms were compared. The frequency shift for the pig blood-glucose liquid phantoms showed a linear increase, whereas that for the physiological saline-glucose liquid phantoms was nonlinear. Therefore, pig blood-glucose phantoms were deemed more suitable for experiments. Kandwal et al. proposed a spoof surface plasmon polariton (SSPP) endfire sensor for sensitive glucose detection [85]. The sensor radiates an endfire beam into the sample with a considerably reduced effective aperture. An additional pair of triangular ground planes located at the CPW port of the sensor suppress sidelobes that interfere with accurate glucose detection, and the slow wave of the SSPP endfire sensor increases the sensitivity as shown in Figure 10a. As a result, the resonant frequency of the sensor increases as the glucose concentration increases from 75 mg/dL to 150 mg/dL as shown in Figure 10b.

**Figure 11:**
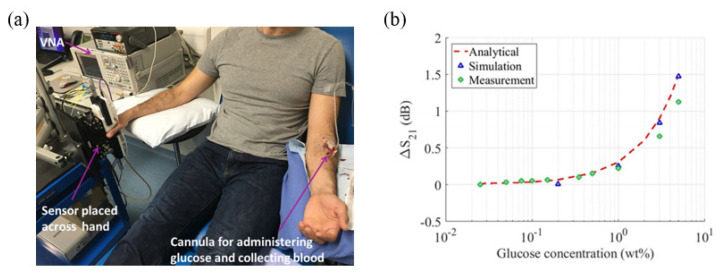
Glucose concentration detection using a 60 GHz microstrip patch antenna. (**a**) Experimental setup for a real-time in vivo human clinical test. (**b**) Variation in the transmission coefficient of the sensor as a function of the glucose concentration [86].

A glucose sensor based on two microstrip patch antennas operating at 60 GHz has also been proposed [86]. The proposed sensing system consists of two facing antennas placed across the sample. As the dielectric properties vary due to changes in the glucose concentration, the transmission between the two microstrip patch antennas also changes. A real-time in vivo human clinical test was conducted to investigate the sensor response as shown in Figure 11a. Thus, glucose concentrations from 0.025 wt% to 0.5 wt% can be detected based on variations in the transmission coefficient as shown in Figure 11b.

### 4.5. Planar Resonator-Type Glucose Sensors

Microwave resonators are lightweight, cost-effective, easy to fabricate, portable, and reusable and have low power consumption and low profiles [87]. Various microwave resonators have been the most commonly used devices as sensors in recent decades because of these advantages. For example, a temperature sensor using a surface acoustic wave (SAW) resonator [88], a nitrogen dioxide ($NO_{2}$) and ethanol gas sensor based on a double split-ring resonator (DSRR) [89], a breast tumor sensor based on a dielectric resonator [90], a sensor for detecting cancer biomarkers using film bulk acoustic resonators (FBARs) [91], a sensor for biomolecule detection using a split-ring resonator (SRR) [92], a relative humidity sensor based on a DSRR and an active resonator [93,94], a pH sensor based on a hexagonal split-ring resonator (HSRR) [95], a sensor for streptavidin detection based on an open-loop resonator [96], a vital sign sensor based on a rectangular resonator [97], a sensor for cardiorespiratory sign detection using a complementary split-ring resonator (CSRR) [98] and a wrist pulse sensor based on array resonators [99] have been reported. Due to their need for only a small sample volume and their many other advantages, microwave resonators are widely used for glucose detection.

Kim et al. measured glucose solutions with different concentrations from 0 to 300 mg/mL by using a quarter-wavelength dielectric resonator with a gap for sample placement [100]. The resonant frequency of the dielectric resonator was 1.68 GHz, and the glucose concentration was detected based on the shift in the resonant frequency and the reflection coefficient. They analyzed the effect of sample volumes from 1 μL to 5 μL, and the results showed that the frequency shift increased with the sample volume. Additionally, both the reflection coefficient and the resonant frequency shift increased as the glucose concentration increased. Odabashyan et al. designed a resonator based on a modified first-order Hilbert curve to detect glucose concentrations of 0–250 mg/dL in steps of 50 mg/dL at a resonant frequency near 6 GHz [101]. The electromagnetic near field of the resonator was analyzed through simulation, and a glass container containing a glucose solution of 500 μL was placed on the surface where the near field was the strongest. The sensing parameter was the transmission coefficient, which decreased as the glucose concentration increased. In another study, glucose solutions with different concentrations of 0–5 mg/mL were detected by means of a complementary split ring resonator (CSRR) at 2.48 GHz [102]. The glucose solutions were flowed through a microfluidic channel located in a sensing area made of polydimethylsiloxane (PDMS). When the glucose concentration changed, the capacitance between two copper metal pieces also changes, causing changes in the reflection coefficient and resonant frequency. The measured data were located in region A of the Clarke error grid, where the error is less than 20%, indicating accurate sensor performance. Choi et al. proposed a glucose sensor based on discrete double split-ring resonators with an aluminum shield that is robust against interference from temperature fluctuations [55]. They simulated the penetration depth of the electric field into an abdominal model composed of skin, fat, muscle, small intestine and bone layers. The simulated results showed that the electric field rapidly decays in the skin and muscle layers because of their high moisture content. The two discrete ring resonators have different resonant frequencies. One is affected by the glucose concentration, and temperature fluctuations cause the other to change. By considering the two resonant frequency shifts, temperature variations can be calibrated out. The performance of the sensor was demonstrated in a test of continuous monitoring over 12 h by comparison with a commercial continuous glucose meter.

**Figure 12:**
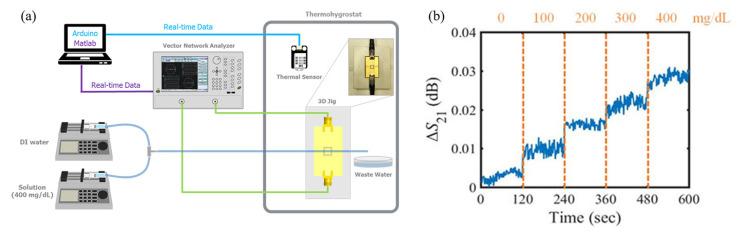
Glucose concentration detection using a complementary split-ring resonator with temperature correction. (**a**) Experimental setup for continuous measurement of glucose concentration. (**b**) Variation in the transmission coefficient of the sensor as a function of the glucose concentration after temperature correction [103].

Jang et al. detected glucose solutions using a CSRR with a resonant frequency of 2.42 GHz [103]. For different concentrations and temperatures, the dielectric constant and loss tangent of the glucose solutions were measured using an open-ended coaxial dielectric probe. It was verified that the dielectric properties were more significantly affected by temperature than by concentration. Thus, elimination of the temperature effect was necessary for accurate glucose detection. To this end, the variation in the transmission coefficient due to temperature change was measured, and a temperature correction function was derived. The electromagnetic near field was focused on the center of the CSRR, and the fluidic channel hosting the glucose solutions was also located in the center as shown in Figure 12a. Glucose concentrations from 0 to 400 mg/dL in increments of 100 mg/dL were continuously detected without a temperature effect by applying the derived correction function as shown in Figure 12b.

**Figure 13:**
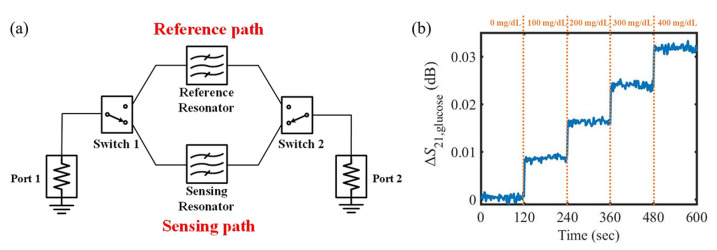
Glucose concentration detection using dual complementary split-ring resonators with a switching circuit for environmental effect elimination. (**a**) Schematic of the glucose sensor. (**b**) Variation in the transmission coefficient of the sensor as a function of the glucose concentration after environmental effect elimination. (Reprinted with permission from [104]; published by IEEE, 2020.)

Our research group developed an improved glucose sensor with environmental effect elimination [104]. The effects of both temperature and relative humidity on the performance of the sensor were analyzed mathematically. The glucose sensor is composed of dual microwave CSRRs and a switching circuit as shown in Figure 13a. One CSRR detects environmental conditions such as temperature and relative humidity, and the other detects both environmental conditions and glucose concentration. By selecting the signal path through the switching circuit every second, the environmental effects are eliminated in real-time. We showed that as a result, glucose concentrations of 0–400 mg/dL in steps of 100 mg/dL can be continuously detected without environmental effects based on the variation in the transmission coefficient as shown in Figure 13b. Compared to the correction function method, the error is reduced considerably.

In addition, many glucose sensors have been reported for the detection of glucose concentration based on resonant frequency shifts [9,10,52,57,105,106,107,108,109,110,111,112,113], variations in the Q-factor [114,115,116], magnitude and phase variations in the reflection and/or transmission coefficient [10,108,114,117,118,119,120,121], and changes in input impedance [54]. The important parameters of various glucose sensors, such as sensor type, operating frequency, the detection range for glucose concentration, sample volume, sample type, sensing parameters, sensitivity, and continuous monitoring capability, are summarized in Table 3.

Accuracy is one of the main factors to evaluate the detection performance. According to International Organization for Standardization (ISO) 15197:2013, blood glucose meter for self-test must satisfy the error within ±15 mg/dL for concentration below 100 mg/dL or ±15% for concentration above 100 mg/dL [122]. The errors of the traditional chemical glucose sensors have a range from 0% to 20% [123]. However, the RF glucose sensors show the error of less than 10% as shown in Table 3.

## 5. Conclusions

In this review, we reviewed the historical landmarks of three generations of glucose sensors and the analysis and measurement of the dielectric properties of glucose solutions in the radio-frequency region. Additionally, various glucose sensors presented in recent decades for noninvasive and continuous monitoring have been reviewed.

However, further research on and development of RF glucose sensors should be conducted to achieve a wholly noninvasive and continuous monitoring scheme for practical use. First, the sensitivity of the sensors should be increased. When the blood glucose concentration changes, this causes only a subtle change in dielectric properties. Thus, it will be necessary to develop a highly sensitive glucose sensor that is robust against external noise and has high accuracy and high stability by optimizing the sensor structure and operating frequency or through integration with an appropriate active circuit system. In addition, proper signal processing techniques are essential for extracting the small signal changes due to glucose concentration variations from the large signal changes due to heartbeat, respiration, temperature variation, etc. Second, the selectivity of glucose sensors must be ensured. In the case of a sensor attached to the skin, blood glucose detection using radio-frequency components is based on the change in the effective dielectric constant, which is a single average value representing several materials with different dielectric constants. Thus, changes in the effective dielectric constant can be induced by variations in multiple tissue components as well as by the blood glucose concentration. To overcome these limitations, techniques such as multisensors using various modalities, big data analysis, and deep learning can be useful.

Microwave engineers and scientists have continuously researched for diverse radio-frequency biosensors for a long time. Unfortunately, the representative commercial RF biosensors with a robust performance have not yet appeared. This is because there are still areas to be solved for the robust RF biosensors, such as optimal frequency, integrated RF devices and circuits, improvement to the signal-to-noise ratio (SNR), discrimination to the other saliva components for RF glucose biosensing. However, since the glucose-sensing research for wireless healthcare monitoring is conducting in technology companies, such as Apple, Samsung Electronics, and Google, we predict that the commercial RF biosensors can also develop as soon as possible soon.

## Figures and Tables

**Table 1:** Historical landmarks in glucose biosensor development (Reprinted with permission from [35]; published by John Wiley and Sons, 2001).

Generation	Event	Reference
1st G	1962-First glucose enzyme electrode	[17]
1st G	1973-Glucose enzyme electrode based on peroxide detection	[36]
1st G	1975-Launch of the first commercial glucose-sensing system	YSI Inc.
2nd G	1982-Demonstration of in vivo glucose monitoring	[28]
2nd G	1984-Development of ferrocene mediators	[26]
2nd G	1987-Launch of the first personal glucose meter	Medisense Inc.
3rd G	1999-Launch of a commercial in vivo glucose sensor	Minimed Inc.
3rd G	2000-Introduction of a wearable noninvasive glucose monitor	Cygnus Inc.

**Table 2:** Dielectric measurements of aqueous glucose solutions via RF spectroscopy [48].

Method	Configuration (Frequency ${}^{1}$)	Reference
Antennas	Wideband monopole antenna (1–6 GHz)	[49]
Antennas	Patch antennas (2.45 GHz, 5.8 GHz)	[50]
Antennas	Patch antennas (5 GHz)	[51]
Antennas	Serpentine-shaped antenna (4.8 GHz)	[52]
Resonator	Open-ended spiral resonator (10 MHz–2 GHz)	[53]
Resonator	Patch resonator (2.45 GHz)	[54]
Resonator	Ring resonator sensor (1.5 GHz)	[55]
Resonator	Resonator combining a spiral inductor and an interdigital capacitor (5.8 GHz)	[56]
Resonator	Cross-coupled stepped-impedance resonator (6.53 GHz)	[57]

^1^ The operating frequency in air.

**Table 3:** Radio-frequency glucose sensors.

Reference	Sensor Type	Frequency (GHz)	Concentration Range (mg/mL)	Sample Volume (μL)	Sample Type	Sensing Parameter	Sensitivity (per mg/mL)	Error (%)	Continuous Measurement
[62]	$TM_{010}$ cavity	3	9–23.4	-	Solution	Frequency	250 kHz	-	X
							Power	9.31 W	
[63]	$TM_{010}$ cavity	3	3.6–25.2	8.8	Solution	Frequency	30 kHz	-	X
							Voltage	0.32 mV	
[64]	$TE_{011}$ cavity	7.542	0.7–1.5	1200	Solution	Frequency	42.875 kHz	-	X
[65]	$TM_{010}$ cavity	2.05	0–36	8000	Solution	$S_{21}$	0.02 dB	-	X
[66]	$TE_{011}$ cavity	4.6	0–3000	-	Solution	$S_{11}$	0.00312 dB	-	X
		+microprobe							
[67]	$TE_{011}$ cavity	4.5	0–10	50	Solution	$S_{11}$	0.004 dB	-	X
		+microprobe							
[68]	Dielectric waveguide	2.15	0–50	1000	Solution	$S_{11}$	0.0028 dB	-	X
		+microprobe							
[73]	SIW	5.8	1–5	-	Solution	Frequency	24.05 MHz	-	X
[75]	$TM_{010}$ circular SIW	4.4	0–300	2.5	Solution	Frequency	383 kHz	±0.44	X
							$S_{21}$	0.013 dB	
[80]	Patch antenna	5.5	0.5–5	-	Solution	Frequency	39.25 MHz	-	X
		+SIW slot antenna							
[81]	Two planar	3–10	0–40	-	Phantom	$S_{21}$	0.14 dB	-	X
		UWB slot antennas							
[82]	U-shaped antenna	1.9	0–40	40,000	Solution	Frequency	1.25 MHz	-	X
							$S_{11}$	0.5 dB	
[83]	Inset-fed	2.4	1–5	-	Solution	Frequency	7.5 MHz	-	X
		patch antenna							
[84]	Patch antenna	1.36	1–3.5	-	Blood	Frequency	500 MHz	-	X
[51]	Inset-fed	5	0–5	25,000	Blood	Frequency	17.2 MHz	-	X
		patch antenna							
[85]	SSPP endfire antenna	8.9	0.75–1.5	-	Blood	Frequency	150 MHz	-	X
[86]	Patch antenna	60	0–5	-	Blood	$S_{21}$	0.025 dB	10	O
[100]	Dielectric resonator	1.68	0–300	4	Solution	Frequency	16.8 kHz	-	X
							$S_{11}$	0.003 dB	
[101]	Hilbert-shaped	6	0–2.5	500	Solution	$S_{21}$	1.56 dB	±2	X
		resonator							
[102]	CSRR	2.48	0–5	-	Solution	Frequency	500 kHz	3.3	X
							$S_{11}$	0.6 dB	
[55]	Discrete DSRR	4	0–7.2	21,000	Blood	Frequency	18.24 kHz	0.5	O
[103]	CSRR	2.42	0–4	3.9	Solution	$S_{21}$	0.0075 dB	-	O
[104]	CSRR	2.42	0–4	3.9	Solution	$S_{21}$	0.008 dB	-	O
[105]	SRR	4.2	0–50	-	Solution	Frequency	2.6 MHz	-	X
[52]	Resonator	4.8	0–20	120	Solution	Frequency	1.6 MHz	3	X
[106]	Rectangular meander	9.2	0–5	1	Blood serum	Frequency	92.6 MHz	1	X
		line resonator							
[57]	Stepped-impedance	6.53	0–5	2	Blood serum	Frequency	978.7 MHz	2.4	X
		resonator							
[107]	SRR	2	10–150	-	Solution	Frequency	10.5 kHz	-	X
							Amplitude	0.0017 dB	
[108]	Air-bridge-type	1.5	0.3–5	0.1	Solution	Frequency	117.5 kHz	1.1	X
		LC resonator					$S_{11}$	0.49 dB	
[109]	SRR	1.61	0–400	90	Solution	Frequency	174 kHz	7.3	O
[110]	SRR	1.61	0–500	90	Solution	Frequency	107 kHz	7.3	O
[10]	Chipless tag	4	0–7.2	200	Phantom	Frequency	210.92 kHz	-	O
		SRR					$S_{21}$	0.0084 dB	
[111]	Complementary	1.16	0–100	-	Solution	Frequency	2.11 MHz	-	X
		electric-LC resonator							
[112]	Complementary	1.71	0–500	95	Blood	Frequency	1.85 MHz	-	X
		electric-LC resonator							
[9]	Four-cell	2	0.4–1.4	600	Solution	Frequency	95 MHz	-	O
		hexagonal CSRR							
[113]	CSRR	5.41	0–80	100	Solution	Frequency	6.25 MHz	-	X
[114]	Open-loop	5.16	0–100	25	Solution	Q-factor	0.0658	2	X
		microstrip resonator					$S_{21}$	0.0084 dB	
[118]	LC resonator	3.41	40–200	20	Solution	$S_{21}$	0.053 dB	-	X
[119]	Interdigitated	7.5	0–80	-	Solution	$S_{21}$	0.0076 dB	-	X
		capacitor							
[120]	Ring resonator	4.01	0–2.5	-	Solution	Phase($S_{11}$)	0.2 deg	-	X
							Phase($S_{21}$)	0.48 deg	
[121]	Epsilon-negative	2.074	0–100	-	Solution	Voltage	1.4 mV	1.41	X
		resonator							

## Data Availability

Not applicable.
